# Reinventing the human tuberculosis (TB) granuloma: Learning from the cancer field

**DOI:** 10.3389/fimmu.2022.1059725

**Published:** 2022-12-15

**Authors:** Senait Ashenafi, Susanna Brighenti

**Affiliations:** ^1^ Department of Medicine Huddinge, Center for Infectious Medicine (CIM), Karolinska Institutet, ANA Futura, Huddinge, Sweden; ^2^ Department of Pathology, School of Medicine, College of Health Sciences, Tikur Anbessa Specialized Hospital and Addis Ababa University, Addis Ababa, Ethiopia

**Keywords:** tuberculosis, *Mycobacterium tuberculosis*, granuloma, immunity, macrophage, T cell, cancer, immune checkpoint inhibitor (ICI)

## Abstract

Tuberculosis (TB) remains one of the deadliest infectious diseases in the world and every 20 seconds a person dies from TB. An important attribute of human TB is induction of a granulomatous inflammation that creates a dynamic range of local microenvironments in infected organs, where the immune responses may be considerably different compared to the systemic circulation. New and improved technologies for *in situ* quantification and multimodal imaging of mRNA transcripts and protein expression at the single-cell level have enabled significantly improved insights into the local TB granuloma microenvironment. Here, we review the most recent data on regulation of immunity in the TB granuloma with an enhanced focus on selected *in situ* studies that enable spatial mapping of immune cell phenotypes and functions. We take advantage of the conceptual framework of the cancer-immunity cycle to speculate how local T cell responses may be enhanced in the granuloma microenvironment at the site of *Mycobacterium tuberculosis* infection. This includes an exploratory definition of “hot”, immune-inflamed, and “cold”, immune-excluded TB granulomas that does not refer to the level of bacterial replication or metabolic activity, but to the relative infiltration of T cells into the infected lesions. Finally, we reflect on the current knowledge and controversy related to reactivation of active TB in cancer patients treated with immune checkpoint inhibitors such as PD-1/PD-L1 and CTLA-4. An understanding of the underlying mechanisms involved in the induction and maintenance or disruption of immunoregulation in the TB granuloma microenvironment may provide new avenues for host-directed therapies that can support standard antibiotic treatment of persistent TB disease.

## Introduction

*Mycobacterium tuberculosis* (Mtb) is an intracellular bacterium with a remarkable ability to manipulate immune pathways in the human host to escape from the local defense in the lung or other infected organs. The hallmark of human tuberculosis (TB) is the formation of granulomas, which is defined as aggregates of immune cells, particularly Mtb-infected macrophages in response to chronic inflammation. The typical morphology of the TB granuloma is well-established including a core of modified macrophages such as epithelioid cells, foamy macrophages and multinucleated giant cells and more or less organized layers of lymphocytes and granulocytes with varying degrees of fibrosis and caseous necrosis (1). However, these structures are highly dynamic and less is known about the role of the granuloma microenvironment (GME) in regulating immune cell function and controlling bacterial growth. While it is widely accepted that cell-mediated immunity including activated macrophages and IFN-γ producing T cells is imperative to achieve immune control of intracellular Mtb, it is debated whether immunoregulatory subsets such as anti-inflammatory macrophages, myeloid-derived suppressor cells (MDSCs) and regulatory T cells (Tregs) are required to dampen chronic pathological inflammation or if the induction of immunosuppressive cells contribute to an impaired antimicrobial effector response. This controversy has escalated by the findings that cancer patients who receive therapy with immune checkpoint inhibitors targeting the PD-1/PD-L1 pathway, seem to have an increased risk of reactivating latent TB infection (2, 3). Immune checkpoint inhibition has in many ways revolutionized cancer therapy by blocking primarily the inhibitory molecules, PD-1 or CTLA-4, that are expressed by exhausted effector T cells or Treg cells (4). In this Review, we will attempt to provide a deepened view on the most recent findings of selected *in situ* studies related to the GME and how we can learn more about the phenotype and function of the GME by comparisons to studies on the tumor microenvironment (TME). Interestingly, TB granulomas share many similarities with solid tumors, including tissue remodeling, regions of hypoxia and necrosis, extensive fibrosis, and local immunosuppression (5). Our intention is not to provide a broad overview on the GME, but to touch base with some of the most recent studies using multiparametric imaging methods and single-cell RNA sequencing (scRNA-seq) to discover the different micro-milieus of the granuloma lesions with a focus on human and primate tissues. We believe this is important to enhance our understanding about protective as well as non-protective immune mechanisms identified in the GME and in what way we can manipulate the human immune response at the site of Mtb infection with relevance for host-directed or immune enhancing therapies in TB.

### Innate and adaptive immune cells as key players in cancer and TB immunity

**Figure 1:**
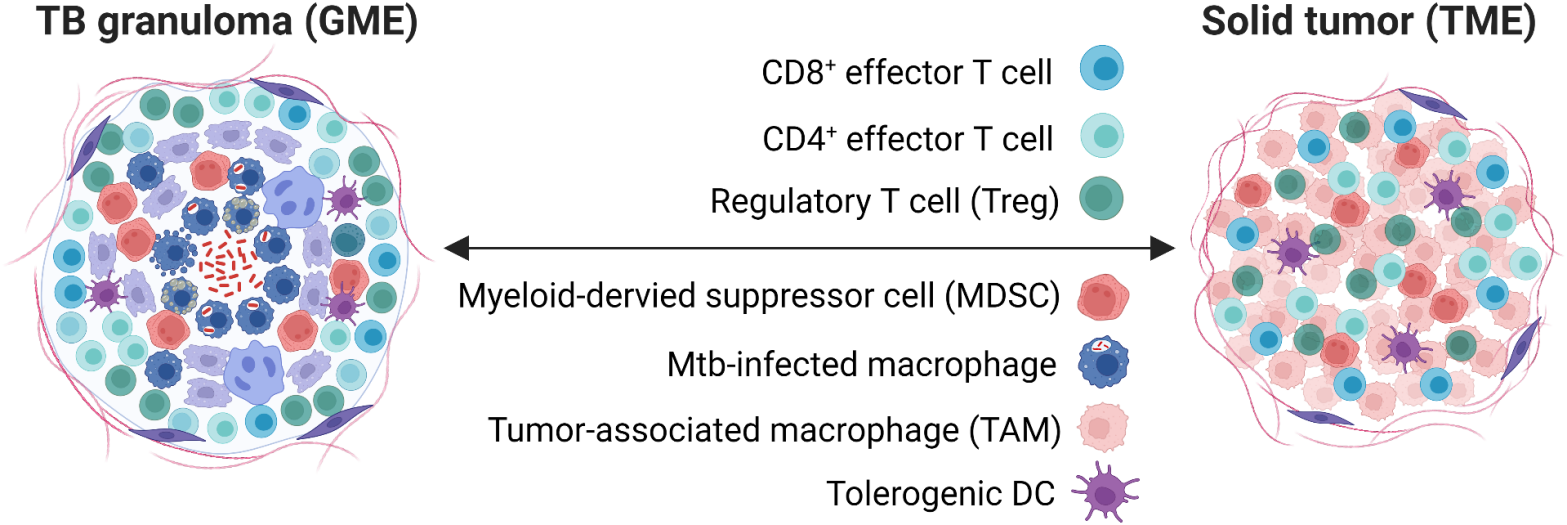
The TB granuloma versus solid tumor microenvironment. Shown are relevant immune cells involved with a focus on myeloid cells and T cell subsets, several with immunosuppressive functions that are controlled by checkpoint molecules. Light purple cells in the TB granuloma depict epithelioid cells and multinucleated giant cells, while dark purple cells depict fibroblasts. GME, granuloma microenvironment; TME, tumor microenvironment.

The immune microenvironment is crucial for the prognosis and outcome of localized diseases that require cell-mediated immunity including chronic infections and different tumor types. The importance of the immune response in preventing active TB as well as cancer is highlighted by the increased frequency of clinical TB (6–8) and different malignancies (9–11) in immunosuppressed and immunodeficient patients. Immunologically, granulomatous TB lesions are in many ways similar to tumors (**Figure 1**) in that immune cells are persistently exposed to specific antigens and bystander stimuli that result in potent inflammation and immune cell activation, but also exhaustion, functional inactivation or local immunosuppression (12–14). Antigen-specific T cells are found at the local site of disease and in the peripheral circulation of patients. A central role of both conventional and unconventional CD4+ and CD8+ T cells have been described in TB infection (15) and cancer (16) including complementary as well as overlapping functions of CD4+ and CD8+ T cells (17). CD4+ T cells are considered mandatory to support activation of other immune cells such as macrophages, CD8+ T cells and B cells by production of Th1 effector cytokines IFN-γ and TNF-α (18). Instead, CD8+ T cells are primarily involved in contact-dependent killing of target cells either *via* death receptor/ligand ligation or granule-mediated exocytosis of cytotoxic effector molecules such as perforin, granzymes and granulysin (19, 20). However, under specific circumstances CD4+ T cells are activated to express cytotoxic killing functions and correspondingly, CD8+ T cells are important producers of Th1 cytokines that can stimulate immune cells present in the local granuloma or tumor microenvironment (18). As such, Th1 cells are responsible for activation of cell-mediated immunity that is required to combat intracellular pathogens and malignant cells (17, 21). This involves activation of dendritic cells (DCs) and macrophages, but also persistence of cytotoxic natural killer (NK) cells and cytotoxic CD8+ T cells (CTLs) as well as Th1 effector CD4+ T cells (13, 22). In contrast, Th2 cells producing IL-4, IL-5 and IL-13 are associated with activation of granulocytes involved in allergic reactions as well as activation of B cells and humoral immunity that are usually not effective in intracellular infections such as Mtb nor in anti-tumor responses (21). Similarly, immune inhibition mediated by MDSCs, anti-inflammatory macrophages and Treg cells may skew the intended tissue repair response towards active immunosuppression at the local site of disease (13).

Innate and adaptive cells involved in cellular immune responses communicate to recognize and control or destroy Mtb-infected cells and cancer cells. NK cells and gamma-delta (γδ) T cells are innate lymphocytes that have been shown to be of importance in the early defense against infected cells (23, 24) or tumor cells (25, 26). Mtb-infected macrophages or tumor-associated macrophages (TAMs) derive from both tissue-resident cells and from peripheral blood monocytes that are recruited to the disease site in response to inflammation (27). Th1 cytokines including IFN-γ, promote polarization of classically activated M1 macrophages that secrete pro-inflammatory cytokines such as IL-1β, IL-6 and TNF-α and have potent antigen processing and presenting abilities as well as cytotoxic and antimicrobial activities and are considered anti-tumorigenic (28, 29). Proinflammatory M1 macrophages are capable of killing Mtb-infected cells or tumor cells *via* release of lysosomal enzymes, reactive nitrogen intermediates (RNI) and reactive oxygen species (ROS). In contrast, Th2 or anti-inflammatory cytokines including IL-4, IL-10 and TGF-β, polarize alternatively activated M2 macrophages that express arginase-1 and have poor cytotoxic activities but are instead associated with wound healing processes (29, 30). Anti-inflammatory M2 macrophages support angiogenesis and extracellular matrix remodeling and are involved in suppression of Th1 and CTL responses (31). Alveolar macrophages are the main host cell to become infected with Mtb in the early stage of infection (32) and bacteria are likely allowed to replicate in these cells because of their inherent anti-inflammatory or tolerant function in lung homeostasis (33). Polarization of anti-inflammatory or immunoregulatory macrophages may be considered an immune evasion mechanism used by virulent strains of Mtb to promote long-term persistence in the GME (34–37). TAMs are the most abundant population of tumor-infiltrating immune cells in the TME (38) and typically belong to the M2-like macrophage population (29, 39) that are associated with poor prognosis in different cancers (40, 41). Accordingly, M2-polarized macrophages are permissive to intracellular Mtb growth (34, 42) and also promote tumor growth and metastasis (27). It is worth noticing the emerging data uncover the M1/M2 classification as an oversimplification of macrophage polarization. The complexity of monocytes/macrophages in the tissue microenvironment *in vivo* show a poor correlation with the M1/M2 designation, which suggest that distinct populations play specific roles in Mtb infection or tumor progression (33, 43–45).

Modern single-cell sequencing and tracer technologies have enabled identification of diverse subsets of macrophages in tissues such as the lung with relevance for different disease conditions (33, 43). These include tissue-resident and monocyte-derived alveolar macrophages as well as interstitial macrophages in Mtb-infected lungs (46, 47) and several functionally distinct monocyte/macrophage populations that predicted the prognosis of lung adenocarcinoma (48). Recent evidence also points to a role of inflammation-driven metabolic reprogramming of macrophages that contribute to the regulation of immune functions in the GME (49). It was recently reported that Mtb infection of macrophages can prevent glycolysis in pro-inflammatory macrophages and limit metabolic reprogramming over time to favor bacterial growth (50). Correspondingly, inhibition of fatty acid oxidation, promoted antimicrobial functions and reduced Mtb replication in macrophages (51). Likewise, a key mitochondrial fatty acid β-oxidation enzyme was recently shown to be involved in progression of aggressive prostate cancer including enhanced risk of metastasis and poor clinical patient outcome (52). Overall, macrophages have a key role in balancing the inflammation in the GME as well as the TME involving a combination of pro- and anti-inflammatory responses to combat Mtb-infected macrophages and tumor cells and to control tissue pathology as is also discussed below (53, 54). However, up to date there is no coherent consensus around macrophage heterogeneity and classification and the role of specific subsets at sites of disease and during different phases of disease.

### Immune features in the TME that resembles the GME: Local immunosuppression mediated by myeloid cells and regulatory T cells

Treg cells are usually represented by a heterogenous sup-population of CD4+ T cells with a specialized function in suppression of effector cells *via* cell contact-dependent mechanisms or secretion of anti-inflammatory cytokines such as IL-10, TGF-β and IL-35 (55, 56). Although Treg cells have a protective function to reduce pathological inflammation in tissue, these cells also have an inhibitory effect on specific anti-Mtb as well as anti-tumor responses. Different factors such as immature or tolerogenic DCs or high TGF-β or IL-10 production may drive expansion of Treg cells (56), which could have an unfavorable impact on the local ratio of Treg to effector T cells in the TME (57, 58) and in the GME (59, 60). A typical trait of chronic infections and cancer is exhausted T cells in the local microenvironment that lose robust effector functions, express multiple inhibitory receptors and are defined by an altered transcriptional program (14). Such dysfunctional T cells express diverse sets of inhibitory receptors and/or immunosuppressive cytokines to regulate immunopathology but are often associated with ineffective control of persisting TB infection (61, 62) and tumors (63, 64). While therapeutic interventions targeting exhausted T cells to restore cellular immunity has been well described in the cancer field, it is debated whether similar approached would be effective in chronic TB infection. Previous findings in the mouse model of TB infection demonstrated that absence of the immune checkpoint molecule PD-1 exacerbated CD4+ T cell mediated immunopathology, which enhanced bacterial load and reduced survival (65). It was recently demonstrated that inhibition of PD-1 in Mtb-infected non-human primates also worsened TB disease, but with a dissimilar T cell pathology compared to mice (66). Granulomas in anti-PD-1 treated primates contained higher bacterial loads and were associated with enhanced levels of pro-inflammatory cytokines and functional CD8+ T cells while CD4+ T cells up-regulated CTLA-4 and displayed reduced trafficking in the GME (66). Other studies have shown that depletion of CD25-postive Treg cells in TB infected mice did not alter the course of infection (67, 68), while depletion of FoxP3-postive Treg cells was shown to ameliorate TB disease (69) and adoptive transfer of CD25+FoxP3+ Treg cells prevented effective CD4+ T cells responses and bacterial clearance (70). These results would suggest that some type of negative regulation of the T cell response to Mtb is required to avoid overt inflammation, however, induction of active immunosuppression in the GME is likely not appropriate to obtain immune protection in TB.

High expression of immune checkpoint molecules such as CTLA-4 on Treg or conventional effector T cells can result in an impaired expression of co-stimulatory molecules on DCs and macrophages and instead promote the expression of the inhibitory enzyme indoleamine 2, 3-dioxygenase (IDO) that result in tryptophan starvation and cell cycle arrest of effector T cells (55, 71). Another enzyme involves the arginase-1 pathway that limits arginine availability for NO synthesis (30) and arginase-mediated depletion of arginine in the local microenvironment may induce a profound suppression of human T cell proliferation and cytokine synthesis (72). MDSCs are a heterogenous population of myeloid cells that expand in response to low-grade chronic inflammation and together with Treg cells contribute to an immunosuppressive tissue microenvironment and disease progression of different infections (5) as well as cancer (73). MDSCs expressing IDO or aginase-1 promote immune tolerance and local immunosuppression in cancer (74, 75) *via* direct inhibition of effector T cells (76) or by expansion of Treg cells (77). Correspondingly, MDSCs have been demonstrated to induce Treg cells in a cell-contact dependent manner (78). Increased frequencies of MDSCs in the lung of TB patients were found to be similar to lung cancer patients, and involved inhibition of proliferation, and cytokine production by CD4+ and CD8+ T cells and modulation of T-cell trafficking (79, 80). Both TB infection (81) and cancer (82) have been associated with an impaired expression of perforin and granzymes in CD8+ T cells, which may result in reduced target cell killing. Interestingly, Treg cells in the TME have also been reported to express granzyme B and perforin that kill NK cells and CD8+ T cells (55, 83), which suggest that Treg cells can suppress granule-associated effector molecules in NK and T cells but also kill effector cells *via* granule-mediated killing.

### Immune features in the TME that resembles the GME: Immunometabolism, vascularization and tissue repair

It has been argued if T cell mediated resistance in chronic infections such as TB would ever be successful in complete eradication of the pathogen, but immune tolerance and control of tissue damage may be as important in host defense in the chronic phase of disease (49). Accordingly, it has been discovered that apart from local immunosuppression, Treg cells have a function in tissue repair and regeneration (84), either indirectly or directly in a tissue-specific manner (85). Excessive inflammation caused by immune cells may result in extracellular matrix (ECM) degradation and tissue injury (37) and subsequent pathological fibrosis that hampers tissue function and may lead to organ failure (85). In the lung, Treg cells inhibit M1 macrophage inflammatory activity and encourage proliferation and differentiation of damaged alveolar epithelial cells (85). Treg cells further promote M2 macrophage polarization *via* the release of anti-inflammatory cytokines (86) that may contribute to the wound healing process but are ineffective in mounting cellular immunity (87). Notably, enhanced levels of granulocytic MDSCs showed an association with low-intermediate chest X-ray findings in patients with active TB, which may suggest a beneficial role of these cells in the limitation of inflammation-induced tissue damage (88). Overall, Treg- or MDSC-mediated suppression of immune cell activity seems to be beneficial for tissue repair and regeneration, but detrimental for maintenance of antigen-specific cellular immunity.

Metabolic pathways (89) and angiogenesis (90) could also influence immune cell function and cellular persistence in chronic infections and cancer by altering the inflammatory processes in the tissue microenvironment. As such, inducible nitric oxide synthase (iNOS) and arginase-1 compete for the same cellular substrate, L-arginine, to produce NO or ornithine, respectively (30). While NO-producing macrophages are microbicidal and anti-tumorigenic, arginase-expressing and ornithine-producing macrophages promote collagen synthesis and tissue-remodeling processes essential for wound healing (30). M2 macrophages have been shown to release anti-inflammatory cytokines and promote angiogenesis and fibrosis (91). Anti-inflammatory M2 macrophages that produce TGF-β promote tissue fibrosis, which may reduce treatment efficacy of standard therapy (12). Hypoxic microenvironments in granulomas as well as solid tumors could also contribute to pro-inflammation and production of vascular endothelial growth factor (VEGF) that promotes angiogenesis and M2 polarization (12). It was previously shown in the zebrafish model that mycobacteria induce granuloma-associated angiogenesis, which promoted bacterial growth and dissemination to new tissue sites *via* permeable blood vessels (92). Likewise, Mtb-infected mice displayed a subpopulation of granuloma macrophages that produced high levels of VEGF that correlated with granuloma size, hypoxia, and necrosis, suggesting that VEGF regulates granulomatous inflammation (93). Studies in Mtb-infected rabbits revealed a functionally abnormal vasculature that only permitted distribution of small molecules in the peripheral regions of the granulomatous lesions (94). Pharmacological inhibition of the VEGF pathway suppressed granuloma-associated angiogenesis and reduced bacterial burden in infected animals (92, 93) likely by normalizing blood vessel formation, which reduced hypoxia and improved small drug transportation (94). Similar to the effect in solid tumors, decreased vascularization during mycobacterial infection increased the T cell to neutrophil ratio in the lesions, which may be considered correlates of protective immunity (95). To regulate inflammation and to promote tissue repair in the chronic phase of TB infection, pro‐inflammatory glycolytic macrophages with antimicrobial properties may skew polarization towards anti‐inflammatory macrophages that rely on oxidative phosphorylation metabolism but lack antimicrobial activities (49). It has been suggested that spatial compartmentalization during granuloma maturation involve such pro‐inflammatory macrophages that attempts to restrict Mtb growth in the granuloma center, while anti‐inflammatory macrophages balance the inflammatory response and limit bacterial dissemination as well as tissue pathology in the granuloma periphery (49, 96). Whether the coexistence of such spatially organized inflammatory and tissue repair responses exist in the human GME or TME remains to be determined.

### The basics of cellular immunity and granuloma formation in human TB

**Figure 2:**
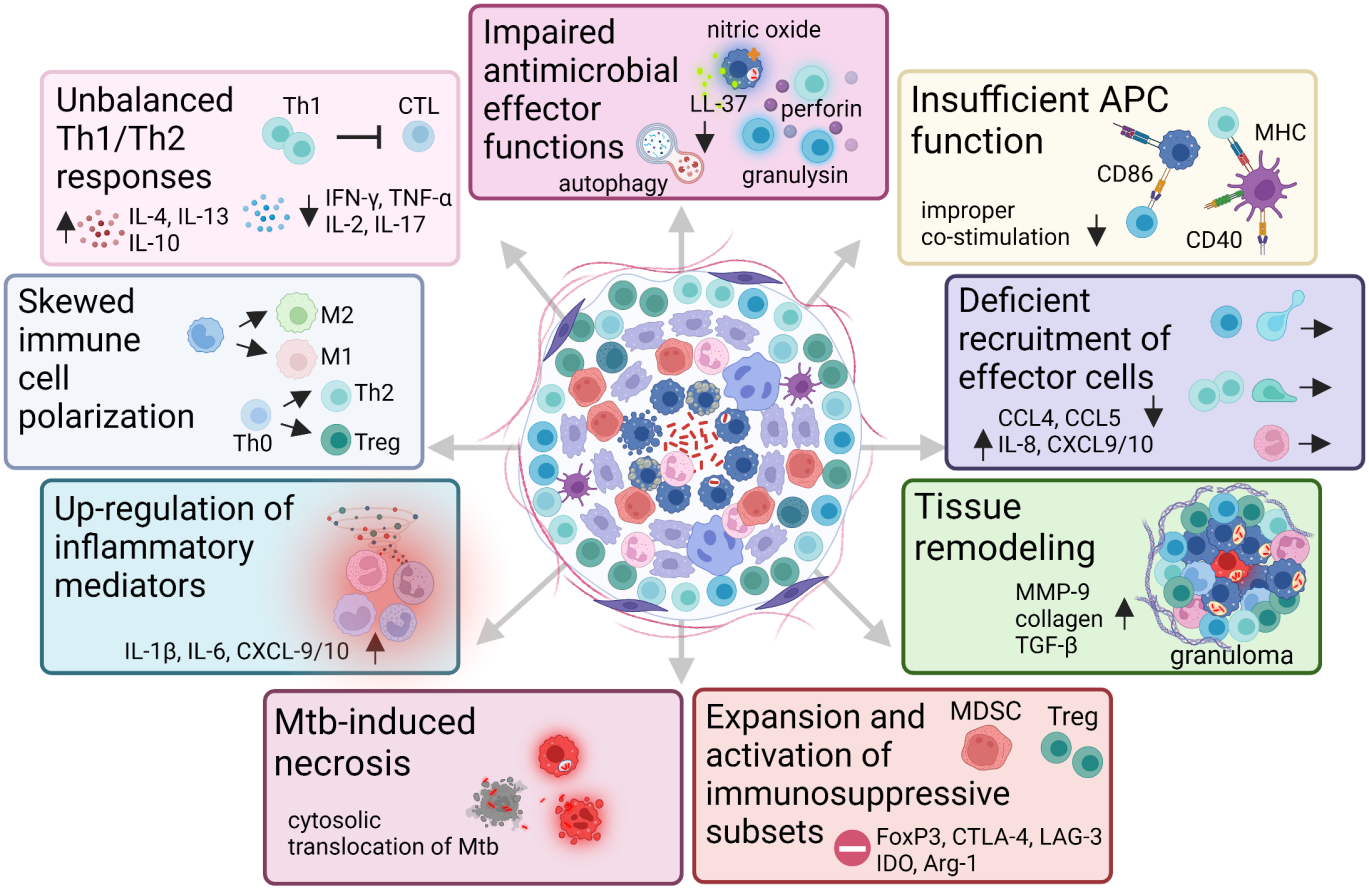
TB control depends on cellular immunity induced in the local GME. Schematic summary of the immune cell alterations and deficient immune pathways that have been described related to induction and maintenance of cellular immunity in the TB granuloma microenvironment. Note that the illustration shows some of the major alterations of cellular immunity in the GME and should not be considered complete.

A schematic outline of the GME and the diverse immune cell pathways in the granuloma milieu that can be altered by virulent Mtb is illustrated in **Figure 2**. The TB granuloma may have diverse functions depending on the phase of Mtb infection. Immature granulomas formed in the early infection phase, may contain cells that are not fully activated and can contribute to seeding of Mtb bacilli from infected macrophages to uninfected monocytes (97). Later on, productive granulomas form that contain modified macrophages such as epithelioid cells and multinucleated giant cells as well as varying levels of immune cell infiltrates including granulocytes and/or antigen-specific T cells with the function to contain and seal-off the infection (98). Small, innate non-epithelioid granulomas may promote bacterial dissemination and seed Mtb infection (97), while formation of mature epithelioid granulomas with varying levels of fibrosis or central caseous necrosis may confine the infection and simultaneously provide a niche for bacterial replication (99). Antimicrobial effector mechanisms in macrophages act as a first-line defense in bacterial clearance involving NO (100), antimicrobial peptides such as human cathelicidin, LL-37 (101), and induction of autophagy (102). Failure of innate immune cells to eradicate Mtb bacilli results in activation and recruitment of adaptive CD4+ Th1 and cytotoxic CD8+ T cells that typically surround Mtb-infected macrophages at the site of infection forming the granuloma (53, 103). Recently, it was demonstrated that help from CD4+ T cells improved CD8+ T cell effector functions and prevented exhaustion, which enhanced intracellular Mtb growth restriction (104). Thus, both CD4+ and CD8+ T cells are required to generate protective T cell responses in TB (104). While CD4+ Th1 cells producing IFN-γ are important to activate macrophages and T cells (105, 106), eradication of Mtb and infected macrophages has been shown to be dependent on CD8+ T cells and granule-mediated killing by perforin and granulysin (20, 107–112). A coordinated expression of Th1 cytokines/chemokines and cytotoxic effector molecules in multifunctional T cells have been proposed to be associated with protective immunity in TB (113) as well as other chronic infections (114, 115). Here, polycytotoxic CD4+ (116) and CD8+ (117) T cells, producing IFN-γ and co-expressing perforin, granzymes B and granulysin, were associated with immune control in persons with latent TB compared to patients with active TB. These studies suggest that effector molecules could cooperate to enter infected cells and kill intracellular pathogens (118, 119), and that not only the magnitude, but the quality of the T cell response is crucial to disease outcome. Importantly, human cytotoxic granules contain the adaptive antimicrobial peptide granulysin, which lack a homologue in rodents such as mice that is a commonly used experimental model to assess TB pathogenesis (120). Mice seems to be more dependent on innate immune responses including NO for control of TB infection (121, 122). Although the importance of CD8+ T cells in protective TB immunity have been demonstrated in different knockout mouse strains (123), there are considerable differences in TB disease pathogenesis comparing different species also regarding the structure and organization of TB granulomas (124, 125). Therefore, the specific model used should be carefully selected and the results obtained should be evaluated against human data. Here, the non-human primate is an important model for human immunology and disease that display features of human TB pathology including Mtb susceptibility and the full spectrum of disease including the various types of pathological lesions observed during human TB infection (126).

Numerous studies provide evidence that TB granulomas have dysfunctional CD4+ and CD8+ T cell responses and restricted infiltration and access of T cells to the central core of Mtb-infected cells where the bacilli reside (60, 127–129). It is not known why T cells are organized in periphery of the granuloma; perhaps as a lymphocytic cuff trying to seal off the infection or as a consequence of deficient T cell migration in the collagen-rich areas of the granuloma. It may also be due to the dynamic tissue repair responses in the peripheral regions of the TB lesions as discussed above. Complete eradication of Mtb in this stage of infection is rare but may instead denote a transition from immune resistance to tolerance and induction of latent or persistent TB (130).

Mtb has evolved successful strategies to undermine cellular immunity, by hiding in infected cells causing down-regulation of effector mechanisms but also blockade of effective antigen-presentation by DCs and subsequent T cell activation including inhibition of crucial chemoattractants and Th1 cytokines (131). In tissue biopsies from lungs or lymph nodes obtained from patients with active pulmonary TB (101, 132) or local TB lymphadenitis (60, 133), respectively, we have observed that severely impaired immune functions involve down-regulation of perforin and the antimicrobial peptides LL-37 and granulysin that are necessary to kill Mtb *via* osmotic lysis. Instead, we and others have shown that persistent Mtb infection promotes expansion of CD4+FoxP3+ Tregs (60, 69, 101, 134), M2-type macrophages (133, 135), or Th2 cells (136, 137), as well as MDSCs (133, 138, 139) with suppressive or anti-inflammatory functions in granulomatous TB lesions. While CD8+ T cells are not necessarily lower in numbers in gross Mtb-infected tissues such as lung or lymph nodes, CD8+ T cells are particularly scarce in the GME (60, 132). The fact that especially CD8+ T cells are low in numbers in the granulomas may be explained by the immunosuppressive microenvironment of the granulomas or by a physical barrier preventing the influx or trafficking of CD8+ effector T cells into the GME. We have also found that the few CD8+ T cells present in the granuloma are functionally impaired with low co-expression of perforin and granulysin, which may suggest that bacterial and host factors in the GME have a negative effect on effector CD8+ T cells. This suggests that Mtb evades cellular immunity and creates a bacteria-permissive environment in the granulomas that may reduce the ability of the host to clear the infection. Whether the different anti-inflammatory or regulatory cell subsets are a cause or consequence of TB disease is unknown.

**Figure 3:**
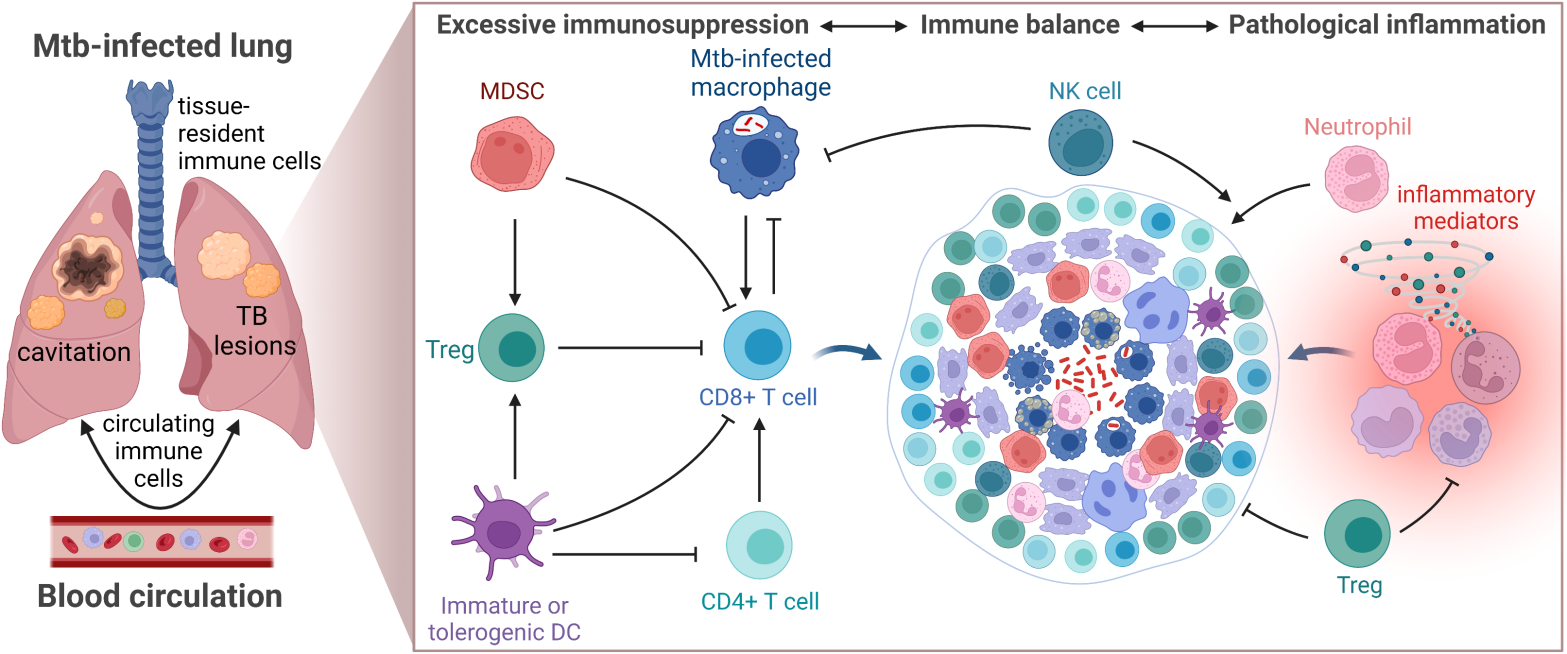
Schematic illustration of the TB granuloma microenvironment. Activation (arrows) and inhibition (closed lines) of immune cells must be strictly controlled to maintain immune balance and prevent TB disease progression.

Poor control of pro-inflammatory and Th1 immune responses promotes pathological tissue destruction and is generally considered to favor TB disease progression (140). However, while excessive inflammation would fuel lung pathology and tissue damage, a too weak response associated with enhanced immunosuppression would result in bacterial replication and spread in the local granuloma microenvironment (**Figure 3**) (141). The question is whether inhibition of immunosuppressive subsets would protect the host or promote pathogen growth? Perhaps more inflammation and effector T cells are not required to eradicate Mtb, whereas restricted but appropriately activated T cells located at the right place in the GME would enhance bacterial killing.

### Recent advances in regulation of TB immunity in the GME

The dynamic nature and complexity of TB granulomas is highlighted by the fact that GMEs with distinct phenotypical and functional properties can exist simultaneously in an infected individual (133, 142). Several studies have been published in the past year aiming to define the different immune landscapes of the Mtb granuloma by mapping the cellular and functional diversity at the local site of infection. New advanced technologies for spatial transcriptomics and high-content imaging have enabled deep-profiling of immune cells in tissue with unprecedented resolution. MIBI-TOF (multiplexed ion beam imaging by time of flight), a multiplex imaging platform that relates cellular phenotype to tissue structure, was used to quantify expression and spatial distribution of 36 proteins in lung tissue sections obtained from patients with pulmonary TB (143). Distinct types of granulomas were identified and defined by structured immune cell compositions and immune cell frequencies that were associated with TB disease status (143). The granuloma composition containing 19 different cell subsets did not differ much comparing pulmonary and extrapulmonary sites, but higher CD8+ T cell frequencies were found in resected lung biopsies from patients who received pre-surgical antimicrobial treatment compared to postmortem lung biopsies (143). This elegant study further demonstrated eight representative microenvironments within the TB granulomas including features of local immunosuppression, such as high expression of the inhibitory enzyme IDO-1 and the immune checkpoint molecule PD-L1 on myeloid cells, but also proliferative Treg cells and high levels of TGF-β in the absence of IFN-γ (143). While most T cell subsets were accumulated in the lymphocytic cuff of the granulomas, CD3+CD4+Foxp3+ Treg cells infiltrated the myeloid-rich core and displayed a positive correlation with IDO+PD-L1+ macrophages that also colocalized with TGF-β (143). Further analyses revealed that PD-L1 expression was associated with active TB progression and treatment response, which suggests that myeloid cells with suppressive properties could promote local expansion of Treg cells and consequently prevent the recruitment and activation of effector CD8+ T cells (143).

Serial intravascular staining was used in combination with intracellular staining and multiparametric flow cytometry to quantify trafficking of blood leucocytes to lung granulomas of Mtb-infected non-human primates (144). Granulomas were found to be dynamic with a slow but continuous cellular influx of primarily T cells and CD11b+ cells, but on average, >90% of the cells from an individual lung granuloma were tissue-localized and not intravascular (144). In contrast to findings in the mouse model of TB, most immune cells and cytokine-producing T cells in TB granulomas remained tissue-localized in primate lungs (144). There was a negative correlation between high bacterial burden and recruitment of immune cells into the granuloma (144). These findings disclose that continual surveillance by relatively low numbers of circulating leucocytes to lung granulomas is important for immune control in TB and that Mtb may restrict recruitment and trafficking of immune cells to the GME. In this context, a systems biology approach using non-human primate data suggested that the structural organization of granulomas as well as recruitment of predominately non-specific T cells likely contribute to reduced responsiveness as <10% of T cells within granulomas have been found to be Mtb-specific in terms of cytokine production (145). There may be several explanations for reduced T cell responsiveness, including exhaustion of T cells in the GME, direct downregulation of antigen presentation by Mtb within infected macrophages, the spatial organization of granulomas that may affect the ability of T cells to reach macrophages and become activated, and/or recruitment of primarily non-Mtb-specific T cells to lung granulomas (145).

Another recent study set out to explore different granuloma trajectories in Mtb-infected non-human primates by comparing the cells and immune pathways involved in high- versus low-Mtb burden granulomas obtained from one and the same infected lung (146). Here, it was suggested that coordinated cell interactions among multiple T cell and macrophage cell subsets are required for successful Mtb control. Combining PET/CT imaging with scRNA-seq of granuloma cells obtained from individual lesions formed early (detected at 4 weeks post-infection) or late (detected at 10 weeks post-infection) revealed that early-appearing, high-burden granulomas were characterized by a type 2 wound healing response driven by IL-4 and IL-13 producing cells and transcripts implicated in fibrosis, TGF-β signaling and tissue remodeling (146). Instead, late-appearing, low-bacterial-burden granulomas were associated with a type 1 response and a higher proportion of a cytotoxic subcluster consisting of conventional αβ CD8+ T cells that expressed genes encoding different effector molecules and functions including perforin and granzymes as well as other genes relevant for motility, migration, tissue residency (CX3CR1, TGFBR3, and S100A10), and regulators of cell state (AHNAK, KLF3, and ZEB2) (146). In addition, bacterial control correlated with CXCR3+CCR6+ T cells with mixed Th1-Th17 traits such as transcripts for TNF-α and IFN-γ, and transcription factors associated with Th17 differentiation (146). These data are consistent with a protective role of Th1-associated T-bet expressed in both CD8+ and CD4+ T cells at later stages of Mtb infection in non-human primates (147). Contrary, FoxP3+ Treg cells expressing CTLA-4, was not associated with bacterial control (146). Importantly, low-burden granulomas reflected more effective bacterial killing rather than reduced bacterial growth, which may indicate that functionally cytotoxic CD8+ T cells could eradicate Mtb-infected cells more effectively in low-burden compared to high-burden granulomas (146). Of note, relatively lower proportions of the cytotoxic CD8+ subcluster was detected in high-bacterial-burden granulomas that showed a skewing towards type 2 tissue repair responses that could inhibit macrophage antimicrobial activity as well as the cytolytic functions of CD8+ T cells in the GME (148, 149). While type 2 responses in early-appearing granulomas have a function to limit pathology, such responses may have the side-effect of preventing and shutting out adaptive T cells that are required for elimination of Mtb-infected cells. Additional information on the spatial distribution of immune cells and granuloma structure in low- versus high-bacterial-burden granulomas may provide additional information on potential protective responses.

Furthermore, scRNA-seq and mass cytometry analyses of immune cells in another non-human primate model of latent versus active TB infection demonstrated that increased lung as well as circulating NK cells expressed cytolytic effectors, including perforin, granzymes and granulysin, which suggest a key protective role for NK-cell mediated cytotoxicity during TB latency in the lung compartment (129). Instead, type I IFN-responsive macrophages as well as plasmacytoid DCs and activated T cells did not seem to contribute to immune control in animals with active TB (129), perhaps because Th1 and Th17 cells are not properly localized close to Mtb-infected macrophages. It is speculated that the early type I IFNs responders is represented by inflammatory monocytes that are recruited from the circulation and differentiate to CD163+IDO-1+ macrophages at the site of infection that recruits Mtb-permissive myeloid cells such as neutrophils and MDSCs, while mediating a T cell suppressive environment in lung granulomas (128). It has previously been shown that IDO-1 expression was particularly high in the macrophage-rich inner layer of TB granulomas that correlated with higher Mtb burden (128). Macrophages co-expressing the M2-marker CD163 together with IDO-1 localized within the suppressive rim of necrotic lung granulomas in non-human primates and humans and may prevent Mtb-specific T cells from reaching infected cells within the necrotic centers, which would promote Mtb persistence (128). Interestingly, inhibition of IDO-1 activity in Mtb-infected primates reduced bacterial growth and lung pathology and allowed translocation and access of granzyme-expressing CD3+ T cells from the peripheral lymphocyte cuff into the core of the granuloma (128). In addition, high expression of the inhibitory molecule LAG-3 in lung granulomas (150), possibly produced by plasmacytoid DCs, may contribute to this immunosuppressive environment that can actively limit T cell protective mechanisms (129).

Altogether, majority of these findings are consistent with our recent report providing evidence that arginase-expressing MDSCs are elevated in TB granulomas from TB/HIV co-infected patients (133). Although we detected high expression of IDO-1 in macrophages in the center of granulomas from TB as well as TB/HIV co-infected tissues, arginase-1 expression was mostly confined to the T cell rich areas localized at the periphery of the TB granulomas, surrounding the cores of the lesions (133). High numbers of granulocytic MDSCs in peripheral blood correlated with Mtb antigen load in tissue, suggesting that enhanced bacterial replication may nurse the expansion of suppressive MDSCs in the granulomas (133). Chronic inflammation coincided with compromised Th1 and CD8+ CTL responses whereas Th2/Treg cytokines ie. IL-13, TGF-β and IL-10, and several inhibitory checkpoint molecules such as IDO, LAG-3 and TIM-3 were enhanced in Mtb-infected tissues, supportive of an immunosuppressive environment in the TB lesions (133). Furthermore, a consistent finding from our *in situ* image analyses clearly show extensive collagen deposition and fibrosis in granulomatous tissue from patients with progressive TB disease (60, 133). An enhanced proportion of granulocytic MDSCs was also found in Mtb-infected non-human primate granulomas, and similar to our observations in humans, the MDCSs were specifically located in the outer lymphocytic cuffs at the periphery of TB granulomas (138). Similar to the potential organization of anti-inflammatory macrophages to the periphery of the granuloma (49), this localization of MDSCs may have a function to restrict T cell access to the core of the TB granuloma and/or contribute to dysfunctional myeloid and T cell responses in the GME. A recent study on SIV/TB co-infected non-human primates found that CD4+ T cells are rapidly depleted from the inner core and outer cuff of lung granulomas and the remaining CD4+ T cells display reduced motility in the GME (151). Furthermore, spatial transcriptomics of 36 markers in granulomas from Mtb-infected C3HeB/FeJ mice, revealed increased levels of Foxp3 and IL-10 mRNA in encapsulated granulomas close to regions with Mtb-infected activated macrophages and high bacterial density (152). Such encapsulated granulomas contained a hypoxic necrotic core surrounded by a thick fibrotic capsule separating the lesion from other lung areas (152). This is also in line with our previous findings from human lymph node granulomas where computerized image analysis demonstrated that CD8+ T cells expressing perforin and granulysin were scarce in the granulomatous lesions while CD4+FoxP3+ T cells co-expressing CTLA-4 and GITR accumulated inside the granulomas together with iNOS-expressing macrophages and high expression of TGF-β (60), suggesting active immunosuppression at the local site of Mtb infection. These studies provide evidence that compartmentalization and skewing of the immune response toward a regulatory phenotype may result in an uncoordinated myeloid and effector T cell response that reduces granule-mediated killing of Mtb-infected cells and subsequently reduce TB disease control.

Recently, scRNA-seq analysis of cells obtained from zebrafish granulomas revealed the expression of mixed type 1- (IFN-γ, IL-12, IL1-β) and type 2-associated transcripts (IL-4 and IL-13), which challenge the current dogma that mycobacterial granulomas exclusively originate from type 1 responses (153). These findings support the notion that macrophage epithelialization and necrotic granuloma formation are dependent on type 2 responses and expression of arginase, while type 1 cytokines and iNOS-expressing macrophages are not involved in epithelioid granuloma formation (153). This type 2-mediated epithelioid transformation and granuloma organization was dependent on down-stream Stat6/IL-4R signaling as zebrafish deficient in these pathways failed to promote epithelialization and necrotic granulomas, which correlated to significantly higher bacterial burden (153). In addition, *in situ* imaging of non-human primate granulomas confirmed abundant presence of arginase-1 expressing epithelioid-like cells in the more distal cell layers from the necrotic core of the granulomas (153). This is also consistent with the spatial compartmentalization of pro- and anti-inflammatory responses associated with tissue repair functions in the GME (49, 96). Instead, these results are in contrast with the above-described study on multimodal profiling of lung granuloma responses in non-human primates (146), which highlights the complexity of the immune milieu in the GME that is also dependent on the experimental model used. More detailed studies on the roles of wound-healing responses and tissue remodeling in TB would shed additional light on these controversies.

### Immune features in the TME that resembles the GME: The cancer-immunity cycle

As described above, chronic TB lesions display multiple similarities with solid tumors including the dynamic changes of macrophage and T cell responses in the local tissue microenvironment as well as regions of hypoxia and necrosis, extensive fibrosis, and local immunosuppression (12–14) (12, 53, 103, 154). Like Mtb-infected macrophages in the GME, TAMs are key cells that can promote an immunosuppressive TME that downmodulates imperative T cell responses (154, 155). Cytotoxic CD8+ T cells are considered to play a major role in granule-mediated killing of cancer cells (156), while a greater focus has been put on CD4+ T cells as well as Mtb-infected macrophages to understand immune control in TB. Although, T and NK cells with cytotoxic functions have been relatively underexplored in Mtb infection, the recent reports described above suggest that CD8+ T cells are important to achieve protective TB immunity in the GME.

**Figure 4:**
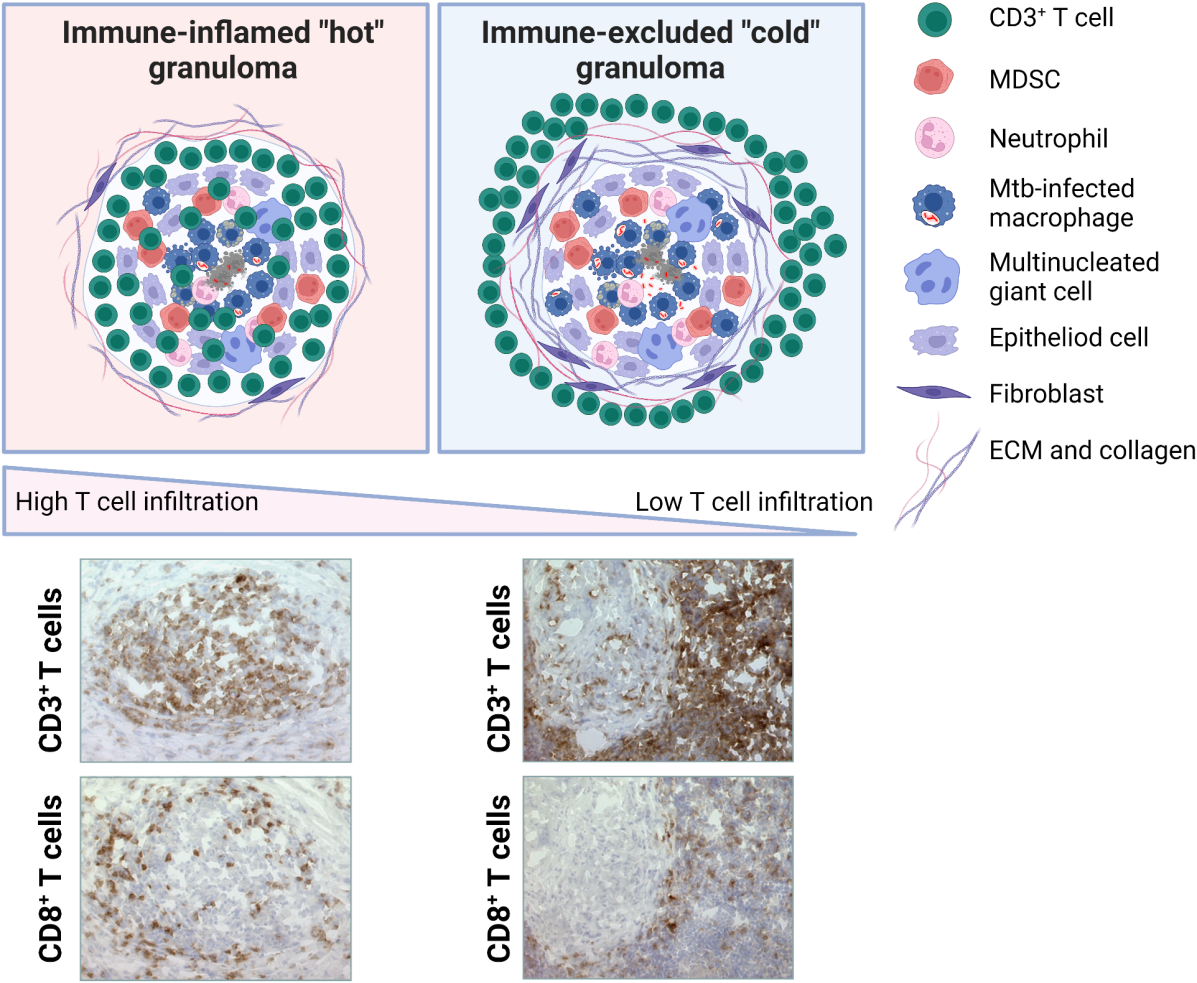
Putative TB granuloma phenotypes. **(A)** Immune-inflamed or hot TB granuloma (left) is characterized by intermediate-high T cell infiltration. These may include both effector T cells and Treg cells at various ratios. Immune-excluded or cold TB granuloma (right) is characterized by low-absent T cell infiltration. T cells are mostly localized around the fibrotic capsule of the lesion. Based on the spatial distribution of CD3+ T cells in the GME, a gradient of these immunophenotypes may be observed. **(B)** Microscopic images illustrating immunhistochemical staining of CD3+ or CD8+ T cells in the GME of selected granulomas from two patients with a local lymph node TB. Representative of immune-inflamed, hot granulomas (left) and immune-excluded, cold granulomas (right). Magnification is x25.

Immune scoring of tumors is built on a conceptual framework involving factors that influence the so-called cancer-immune set point (157). This includes tumor classification into one of three basic immunophenotypes based on the spatial distribution of cytotoxic T cells in the TME: immune-desert, immune-excluded and immune-inflamed phenotypes (from cold to hot tumors) (154, 157). The cancer-immunity cycle is described as a cyclic process that is regulated by a balance between stimulatory and inhibitory signals (154, 157). Below, we mirror the cancer-immunity cycle to the corresponding Mtb infection-immunity cycle as follow: 1.) Release of tumor-antigens/Mtb-antigens, 2.) Presentation of tumor-antigens/Mtb antigens by DCs, 3.) Priming and activation of T cells, 4.) Trafficking of T cells to tumor/granuloma *via* the bloodstream to the disease site, 5.) Infiltration of T cells into tumor/granuloma from the vasculature or periphery, 6.) Recognition of cancer cells/Mtb-infected cells by T cells, 7.) Killing of cancer cells/Mtb-infected cells by cytotoxic T cell destruction *via* granule exocytosis (perforin, granzymes and/or granulysin) (154, 157, 158). Dying tumor/Mtb-infected cells release additional antigens, allowing the tumor/infection-immunity cycle to continue. Notably, tumors with the immune-desert phenotype cannot pass steps 1-3 due to the absence of T cells in both the tumor and its margins. Tumors with the immune-excluded phenotype cannot exceed steps 4-5 due to a lack of T cells in the tumor mass. Tumors with the immune-inflamed phenotype cannot exceed steps 6-7 due to T cell exhaustion or immunosuppression due to Tregs and/or MDSCs and up-regulation of immune checkpoint inhibitors such as PD1-PD-L1, CTLA-4 or IDO. Whether the cancer-immunity cycle can also be applied to Mtb infection is yet to be determined, but it is also possible that TB immunity follows separate paths or branches where the granuloma immunophenotypes exist as diverse entities in the infected organ. The schematic in **Figure 4**, illustrate the conceptual notion of hot or immune-inflamed versus cold or immune-excluded TB granulomas that like hot and cold tumors, exhibit varying degrees of T cell infiltration. Occasionally, the term hot and cold granulomas have been used when referring to the level of bacterial replication or metabolic activity (159), but here we use this term to define the relative infiltration of T cells into the infected lesion.

In a similar model that is based on the distribution and number of CD3+ and CD8+ T cells in the center and edge of tumors and also on the expression level of PD-L1, tumors are divided into hot, cold, and intermediate tumors (including immune-suppressed and isolated) (160). Immune evasion mechanisms in tumor immunology involves factors that inhibit T cells and T cell recruitment, which prevents cytotoxic T cells from entering the TME (160). In immune-desert tumors, CD8+ T lymphocytes are absent from the tumor and its periphery, while in immune-excluded tumors, CD8+ T cells localize only at invasion margins and do not efficiently penetrate the tumor. Inflamed tumors (158) are featured by the presence of T cells in the tumor parenchyma or at the peripheral margin of the tumor (161), but these may consist of both effector and regulatory subsets to maintain immune homeostasis (162). This phenotype also contains proinflammatory cytokines and chemokines that could benefit T cell activation, expansion, and cellular influx. Contrary, non-inflamed tumors generally express cytokines and Treg cell and/or MDCSs that are associated with immune suppression or tolerance, as well as inactivated or M2-polarized tumor-associated macrophages. Ultimately, the numbers, function, distribution, and migratory abilities of T cells are considered key factors that influence the outcome of tumor development in multiple cancers. Thus, there is a strong incitement to turn cold tumors into hot tumors by improving T cell infiltration that could improve the clinical response to immunotherapy (154). As such, understanding the pathological mechanisms involved in defective T cell migration is essential. Correspondingly, Mtb-induced limitations of granuloma-infiltrating T cells should be further investigated to provide better insights on the potential contribution of T cell-based immunotherapy in TB. Mechanisms that could control T cell migration in cancer as well as TB infection are defects in T cell priming (lack of antigens, insufficient antigen processing/presentation and/or co-stimulation) and deficient T cell homing to the lesions (aberrant chemokines or deficient expression of adhesion molecules, hypoxia) (154). Here, CXCL12 production by fibroblasts or IL-8 production by myeloid cells have been shown to prevent Th1 cells and cytotoxic T cells to enter the tumor parenchyma (163, 164) and also downregulation of the antigen-presentation machinery (164).

### Immune features in the TME that resembles the GME: Extracellular matrix remodeling

Importantly, T cell migration through the tumor stroma is considered a rate-limiting step in the cancer-immunity cycle for the immune-excluded phenotype. The stroma surrounding the tumor parenchyma is enriched in ECM proteins and fibroblasts that may impede the ability of T cells and other immune cells that enter the tissue from the blood, to migrate effectively within this complex environment to reach the tumor cells. A pre-existing anti-tumor response might have been present but was rendered ineffective by a block in tumor penetration through the stroma or by the retention of immune cells in the stroma. This reflects a similar scenario as recently demonstrated in the tertiary lymphoid structures close to the human TB granuloma (143) and could explain the peripheral localization of T cells that is often observed in the GME (60, 127–129). As described above, high TGF-β expression and collagen deposition is characteristic of human TB granulomas and such high-density collagen matrix could likely affect the ability of T cells to reach and kill Mtb-infected cells in the necrotic core of the lesions. Interestingly, cytotoxic T cell release of the granule-associated serine protease granzyme B, can result in degradation of ECM proteins including collagen and promote an immune-inflamed compared to an immune-excluded tumor phenotype (165, 166). There are also other proteases such as the matrix metalloproteinases (MMPs) and CD206+ M2-polarized macrophages that contributes to the ECM degradation, which results in the deposition of a tumor-specific ECM with altered composition and increased stiffness, creating a tumor-supportive environment (167). A matrix-degrading phenotype characterized by elevated expression levels of several MMPs in sputum samples from TB patients correlated with higher TB disease severity scores and a delayed treatment response (168). ECM remodeling and fibrotic activity by ie. fibroblasts in the TME or GME, could therefore emerge as a real threat for the immune response to achieve productive T cell protection as excessive collagen deposition affects the location and migration of T cells that becomes trapped in the fibroblast- and collagen-rich stroma (169). As recently described in human TB, some lung granulomas exhibited a fibrotic wound-healing response including CD36+ fibroblasts and CD163+ M2-like macrophages that were colocalized with collagen-1 within the fibrotic regions of the GME (143). Little is known about the processes that regulate fibrosis in GME including peripheral versus central granuloma-associated fibrosis, although high, local concentrations of TGF-β and IL-10 likely enhance fibroblast proliferation (170). Computational modeling based of human and non-human primate granulomas has also suggested that myeloid cells are drivers of fibrotic disease *via* macrophage-to-myofibroblast transformation (171). While it is a subject of continued debate whether fibrotic granulomas may be beneficial or detrimental to the host, fibrotic granulomas simulated using computational modeling were associated with high bacterial burden (170).

There are several potential drug candidates that can modulate ECM composition to reduce immune-mediated tissue damage and improve TB outcomes and that may be explored as host-directed therapy for TB together with standard antibiotic treatment. Our previous findings in a human organotypic lung tissue model demonstrated that global inhibition of MMPs in the Mtb-infected tissue reduced both granuloma formation and bacterial load (172), suggesting that MMP-targeted intervention could ameliorate TB disease. Accordingly, MMP inhibitors could alter ECM deposition in Mtb-infected tissues and reduce immunopathology and bacterial burden (173, 174). Instead, treatment of Mtb-infected mice with the anti-inflammatory and antifibrotic drug pirfenidone together with the first-line antibiotics rifampin and isoniazid was shown to worsen TB disease, likely by blocking the antibacterial effects of rifampin (175). These findings highlight the importance to study possible drug interactions in different experimental models of TB infection to enhance our understanding about diverse ECM proteins in tissue and the potential beneficial effects from pharmacological interventions targeting ECM remodeling.

### Immune features in the TME that resembles the GME: Relevance of immune checkpoint inhibition

**Figure 5:**
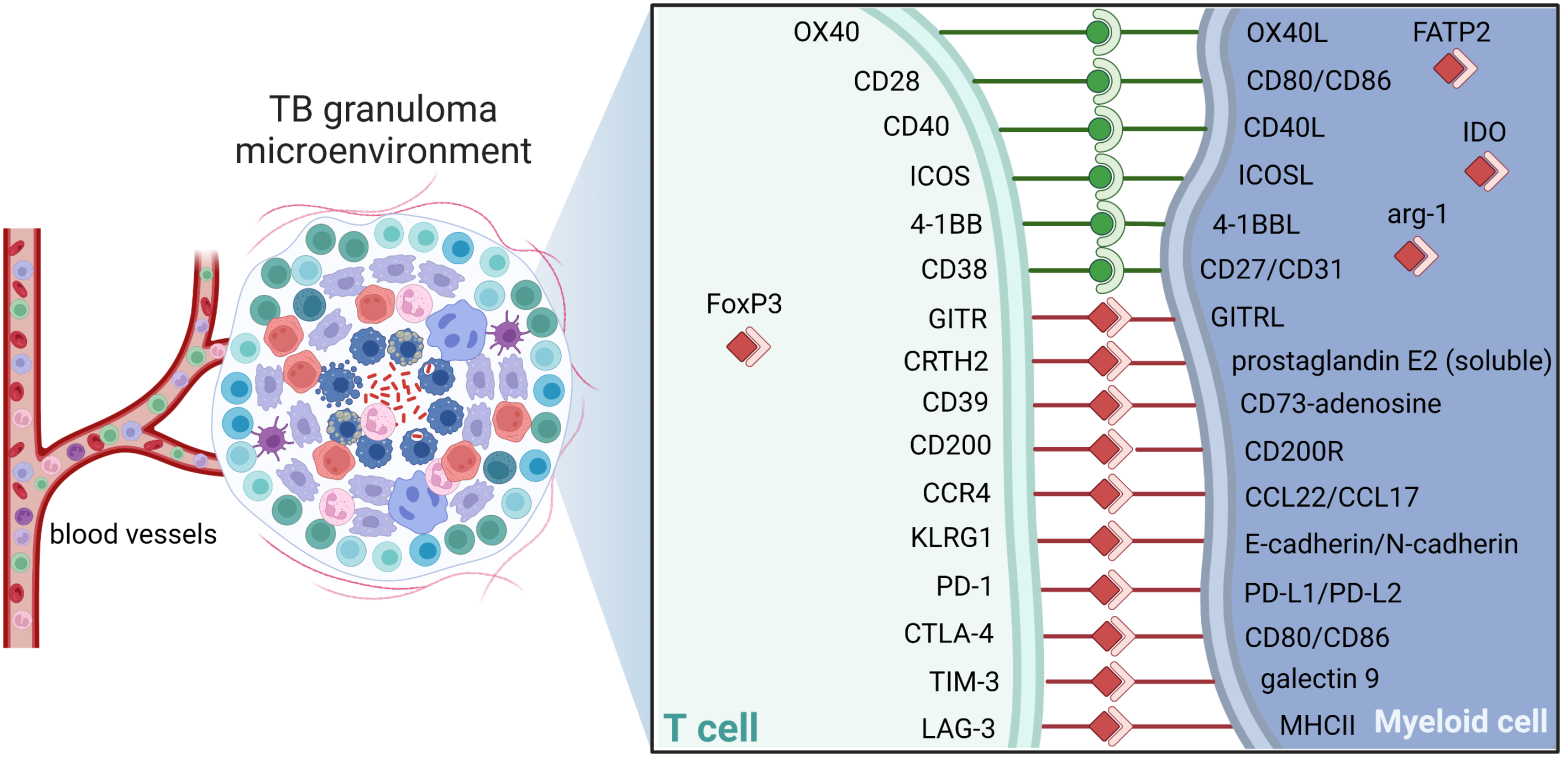
Checkpoint molecules dictate TB control. Schematic of potential immune checkpoint molecules of relevance in TB that can contribute to activation (green) or suppression (red) in the granuloma. Note that selected receptor-ligand pairs are shown and does not represent a global description immune checkpoint molecules. Cell-cell interactions between CD3+ T cells and host macrophages are illustrated. ICOS, Inducible T-cell COStimulator; GITR, glucocorticoid-induced TNFR-related protein; CRTH2, Prostaglandin D2 receptor 2; KLRG1, Killer cell lectin-like receptor subfamily G member 1; CCR4, C-C chemokine receptor type 4; PD1, Programmed cell death protein 1; PD-L1, PD1 ligand; CTLA-4, cytotoxic T-lymphocyte-associated protein 4; TIM-3, T-cell immunoglobulin and mucin-domain containing-3; LAG3, Lymphocyte-activation gene 3; MHCII, major histocompatibility complex II; FoxP3, forkhead box P3; IDO, Indoleamine 2,3-dioxygenase; Arg, arginase; FATP2, Fatty acid transport proteins,.

Understanding the complex structure and immunoregulation of the GME is essential to promote drug accessibility, but also for the development of adjunct immunotherapies that could enhance immune cell functions effective in eradication of persistent bacteria. As in the TME, immune cells in the GME exhibit large phenotypic and functional heterogeneity including expression of both inhibitory and stimulatory immune checkpoint molecules as depicted in **Figure 5** (176, 177). Blockade of checkpoint inhibitors, primarily CTLA-4 and PD-1/PD-1L, has in many ways revolutionized cancer therapy by unleashing effector T cell responses resulting in enhanced tumor cell killing in the local tissue environment (178). Engagement of PD-L1, primarily expressed on antigen-presenting cells, with its receptor PD-1 on T cells delivers a signal that inhibits TCR-mediated activation of IL-2 production and T cell proliferation. As cytolytic T cells are also required to kill Mtb-infected cells, modulation of checkpoint molecules could be considered as adjunct TB treatment (179). However, the effects on checkpoint inhibition or activation on the pathophysiology of Mtb infection is poorly understood. Several case reports show that checkpoint inhibition of T cell subsets using PD-1 blockers reactivates active TB in cancer patients with latent TB (180, 181), which suggest that some of these checkpoints are required to control the immune balance in the GME. Contrary, fewer cases of Mtb reactivation have been reported in cancer patients receiving other checkpoint inhibitors such as PD-L1 (ligand of PD-1) or CTLA-4 (182). Importantly, similar to immunostimulatory treatment with immune checkpoint inhibitors, immunosuppressive treatment such as inhibition of proinflammatory TNF-α in autoimmune diseases, also reactivates TB in latently infected individuals (110). This highlights the complexity in this field and the urge to examine the large heterogeneity of regulatory cell subsets in terms of checkpoint molecule expression to fully appreciate the role of different sub-populations in ongoing, active TB disease (**Figure 5**).

The functions of CTLA-4 and PD-1 seems to be non-redundant, as CTLA-4 has a function to limit early T cell activation in the lymphoid organs, while PD-1 mainly affects T cell activation in the peripheral tissues (176). It has been proposed that inhibition of PD-1 in TB accelerates Mtb growth *via* excessive TNF-α secretion and increased pulmonary TNF-α immunoreactivity (183). Additionally, PD-1 may facilitate host resistance to Mtb by preventing the over-production of IFN-γ by CD4+ T cell that could result in lethal immune-mediated pathology (184). On the other hand, PD-1 and PD-L1 expression on bronchoalveolar lavage-derived CD3+ T cells and CD14+ monocytes obtained from TB patients, suggested that PD-1 was associated with a diminished number of cells producing IFN-γ and TNF-α and IL-2, and blocking the PD-1/PD-L pathway could restores protective T cell responses (185). Furthermore, it was recently discovered that PD-1 blockade induced recovery of dysfunctional PD-1+CD8+ effector T cells but also enhanced immunosuppressive functions of PD-1+ Treg cells, suggesting that the relative proportion of effector PD-1+CD8+ T cells compared to PD-1+ Treg cells in the local TME is very important for the clinical outcome of PD-1 checkpoint inhibition (186). The presence of actively proliferating PD-1+ Treg cells in tumors is therefore a reliable marker for hyperprogressive disease (186). Accordingly, PD-1 blockade may facilitate the proliferation of highly suppressive PD-1+ Treg cells in hyperprogressive cancer, which results in inhibition of effector T cells in tumor tissues (187).

A recent retrospective review that investigated adverse events caused by the group of five FDA-approved PD-1/PD-L1 inhibitors, reported 72 cases of active TB and 13 cases of atypical mycobacterial infections out of a total of 73,886 adverse events (3). Out of the 72 TB cases, 63 were caused by reactivation of latent TB upon treatment with PD-1 inhibitors, while 9 cases were caused by PD-L1 inhibitors (3). Nivolumab, a PD-1 blocking antibody, caused the highest frequency of TB reactivation, while avelumab, which is one of the blocking antibodies of PD-L1, did not cause any events of TB or atypical infections (3). High expression of PD-L1 and IDO1 in the GME (143), also indicates that the efficacy of PD-L1 (or IDO) blockade could differ substantially from PD-1 blockade. In the retrospective study, 44/72 cases occurred in patients with lung cancer that was the most common indication for which use of PD-1/PD-L1 inhibitors leads to TB reactivation (3). Although, TB as a complication in cancer patients treated with PD-1/PD-L1 inhibitors is considered rare, this study found a significantly enhanced risk of TB reactivation in this group of patients (3). Considering around 25% latent TB in any given population, of whom 10% would develop active TB disease, the estimated statistical risk could likely have been higher. Especially because cancer itself is a risk factor for active TB and this patient group are in many ways immunocompromised already before the start of immune checkpoint inhibition. Furthermore, the effects of combination therapy of conventional antibiotics and selected immune checkpoint inhibitors on TB disease outcome is currently not known. Here, it will be important to continue to dissect different immune pathways that could be relevant for checkpoint modulation of both T cell subsets as well as myeloid subsets in the GME and define how the expression of checkpoint molecules are regulated by diverse host or bacterial interactions (**Figure 5**). This is an area of adjunct host-directed therapy that should be further explored in the future using clinically relevant TB disease models.

### Defining disease-specific endotypes that can guide immunotherapy in cancer or TB

Despite the general success of immune checkpoint blockade in human cancers, only 40-60% of cancer patients respond to checkpoint therapy (188). Importantly, the phenotypic immune scores obtained using the cancer-immune set point described above, are becoming particularly important in guiding clinical treatment options as part of precision medicine in cancer. The immune set point is the threshold that must be overcome to generate effective immunity. Thus, a patient with a low set point will be more responsive to ie. immunotherapy, while a high set point will make treatment more difficult. Hierarchical clustering of melanoma patients based on ECM remodeling assessed in liquid biopsies identified three putative endotypes, A-C. Endotype A identified patients with an overall high and differentiated ECM turnover profile who experienced poor outcome when treated with CTLA-4 inhibitors (189). Disease endotypes have recently been described also in TB that can be used to support decision making in future choices of host-directed therapy (190–192). Especially since heterogeneity in TB should not be subtyped simply based on disease phenotype (observable traits such as severity, tissue pathology, and bacillary burden), but diverse endotypes (functional traits based on distinct molecular profiles such as specific metabolic, epigenetic, transcriptional, and immune phenotypes) representing distinct disease states may also require different treatment options (190). As such, TB endotypes can be characterized by either immunodeficiency or excessive pathological inflammation, while other variables such as other co-morbidities or Mtb strain virulence may also drive TB endotypes. Recently, two TB disease endotypes A and B, were determined based on RNA-sequencing profiles of whole blood samples from different TB patient cohorts (191). Endotype A displayed expression of genes related to inflammation and immunity but decreased metabolism and proliferation, while endotype B showed increased metabolic and proliferative activity (191). Precise disease phenotyping, combined with in-depth immunologic or molecular profiling and multimodal omics integration including machine learning models, can identify TB endotypes and guide endotype-specific therapies similar to advances in cancer medicine.

## Conclusions

TB is clearly a multifaceted disease involving a spectrum of unique GME in the infected tissues where bacteria can either persist, thrive, or be killed. While there may be differences in GME structure and composition depending on the stage of Mtb infection and disease progression, a complex mix of diverse GMEs apparently co-exist in the infected host. Recent *in situ* findings suggest a significant and underappreciated role for cytotoxic T cells and/or NK cells in the GME, while immunosuppressive myeloid and Treg subsets likely pose a threat to bacterial elimination. Tissue remodeling involving collagen and other ECM components produced and processed by fibroblasts and anti-inflammatory macrophages likely reduce the antimicrobial capacity of the GME and should be further explored. These studies highlight the need for more knowledge of how to unleash Mtb-specific immunity while limiting inflammatory responses and immunosuppressive subsets aiming to tailor host-directed therapies in patients with different TB endotypes.

The local microenvironment in the TB granuloma resembles solid tumors in multiple ways and therefore we should learn from tumor immune evasion mechanisms to understand loss of immune control and bacterial persistence in granulomas. Defining the factors that influence the TB infection-immune set point at different stages of the infection-immunity cycle or branch, will likely bring us closer to the design of effective immunotherapies that are based on inhibition or occasionally stimulation of checkpoint molecules. Current technological and scientific advances related to *in situ* studies of the TB granuloma tissue offers high-dimensional quantification of both transcriptional and protein data, which follows the expression and distribution of a vast number of molecules at the single-cell level with high resolution. Even though these analyses mostly provide an unbiased snapshot of the cells and immune pathways in a small area of the infected tissues obtained from patients or experimental animals, these data are highly valuable to understand the complexity of the GME and the local factors that may affect altered immune cell morphology and function, tissue remodeling and immune cell migration. Analyses of the GME and Mtb-infected tissues is also an important complement to more easily accessible samples from peripheral blood or body fluids. While blood samples allow for longitudinal as well as functional analyses of diverse immune cell populations, these findings may not always represent the response of immune cells kept in the physiological microenvironment of the infected tissue. Continued research in this area will advance our understanding of regulatory immune cell subsets in different clinical forms or disease-specific endotypes of TB that is necessary to define if modulation of checkpoint molecules would be a beneficial therapeutic approach for difficult-to-treat TB patients.

## Author contributions

SB and SA contributed to drafting the manuscript. All authors contributed to the article and approved the submitted version.
